# Deficient Visuospatial Incidental and Intentional Memory in Cognitively Healthy Middle-Aged First-Degree Descendants of Alzheimer’s Disease Patients

**DOI:** 10.3390/brainsci16040406

**Published:** 2026-04-10

**Authors:** Claudia Guadalupe Álvarez-Huante, María Fernanda Rincón-Ortega, Edgar Josué Palomares-Vallejo, Adriana del Carmen Téllez-Anguiano, Mariana Lizeth Junco-Muñoz, Miguel Ángel López-Vázquez, María Esther Olvera-Cortés

**Affiliations:** 1 Hospital General de Zona No. 1, Instituto Mexicano del Seguro Social, Avenida Bosques de los Olivos No. 101, Colonia La Goleta, Municipio de Charo C.P. 61303, Michoacán, Mexico; klauz_3@hotmail.com; 2 Unidad de Medicina Familiar 84, Instituto Mexicano del Seguro Social, Avenida Hacienda De Chapultepec 395 Morelia, Col. Hacienda Real De La Mina, Morelia C.P. 58332, Michoacán, Mexico; fernandarincon.fr@hotmail.com (M.F.R.-O.); edgarjosue.palomares@gmail.com (E.J.P.-V.); 3 División de Estudios de Posgrado e Investigación, Instituto Tecnológico de México, Avenida Tecnológico 1500, Morelia C.P. 58120, Michoacán, Mexico; adriana.ta@morelia.tecnm.mx; 4 Laboratorio de Neurofisiología Clínica y Experimental, Centro de Investigación Biomédica de Michoacán, Instituto Mexicano del Seguro Social, Camino de la arboleda 300, exhacienda de San José de la Huerta, Morelia C.P. 58341, Michoacán, Mexico; mariana.junco@umich.mx; 5 Facultad de Psicología, Universidad Michoacana de San Nicolás de Hidalgo, calle Francisco Villa 450, Colonia Dr. Miguel Silva, Morelia C.P. 58110, Michoacán, Mexico; 6 Laboratorio de Neuroplasticidad, Centro de Investigación Biomédica de Michoacán, Camino de la arboleda 300, exhacienda de san José de la Huerta, Morelia C.P. 58341, Michoacán, Mexico; migangelv@yahoo.com.mx

**Keywords:** Alzheimer’s disease descendants, incidental visuospatial memory, intentional visuospatial memory, cognitive deterioration

## Abstract

**Highlights:**

**What are the main findings?**

• Cognitively healthy descendants of Alzheimer’s disease patients have deficiency in visuospatial incidental and intentional memory.
• Deficiencies were observed in descendants between the ages of 30 and 55.

**What are the implications of the main findings?**

• Incidental/intentional visuospatial abilities are affected earlier in cognitively healthy Alzheimer’s disease descendants.
• More studies need to assess incidental–intentional visuospatial abilities in this vulnerable population, in order to apply neuroprotective strategies earlier.

**Abstract:**

**Background/Objectives**: Several studies have reported cognitive decline and alterations in neural substrates among non-cognitively impaired direct descendants of Alzheimer’s disease patients. Visuospatial incidental memory declines early in adulthood and may serve as a sensitive tool to detect preclinical deterioration in this vulnerable population. To test this, we characterized the incidental/intentional visuospatial memory of cognitively healthy middle-aged (35–55) descendants of Alzheimer’s disease patients. **Methods**: Male and female first-degree descendants of patients with Alzheimer’s disease (*n* = 55, group DAD) and participants without familial ancestry of the disease (*n* = 55, group NoDAD) were included. Incidental/intentional visuospatial memory was assessed, and the number of correct object–position associations was recorded to classify the participants by accuracy under incidental (4–8, high accuracy) and intentional (6–8, high accuracy) coding conditions. Comparisons with regard to between-group scores and frequency distribution were conducted. A mixed-model design analysis was employed to assess the impacts of the variables on accuracy. **Results**: DAD participants made fewer correct object–position associations than NoDAD under both incidental and intentional coding conditions. In addition, the percentage of NoDAD participants with a high accuracy in both incidental and intentional coding was higher (56.36%) than that for DAD participants (21.81%). A high percentage (36.364%) of DAD participants likewise showed a lower accuracy under both incidental and intentional coding conditions when compared to the NoDAD group (16.364%). Additionally, a marginally significant negative correlation between the number of correct object–position associations and age was found in DAD participants (r = −0.234, *p* = 0.054). **Conclusions**: The present results indicate an impairment in visuospatial incidental/intentional performance related to the familial history of AD, which was seen earlier (35–55 years old, mean age 43.12) in adulthood.

## 1. Introduction

Episodic memory refers to the recording of autobiographical information encompassing the “what”, “where”, and “when” of an event and can be acquired through incidental or intentional coding. Incidental coding means human beings acquire episodic memory without a conscious effort to learn, whereas intentional coding implies a conscient effort to learn (for example, studying). Recall, however, is conscious in both cases [1,2]. Early studies showed that incidental memory is more vulnerable to aging than intentional memory, especially for visuospatial information. Briefly, Naveh-Benjamin, et al. [3] showed that the acquisition of face–name associations but not the memory of individual items (faces or names) was impaired with age. In addition, it was observed that, when acquisition was incidental, older participants were less efficient than young ones in recalling names, faces, and face–name associations, with a more profound deficit in associated information like face–name, word–color, and place–object pairs [3,4,5,6]. This is possibly related to binding processes affected by aging [7].

A similar effect was observed in the decline of spatial memory with age. The vulnerability of visuospatial incidental performance to aging was studied by Uttl and Graef in 1993. The authors performed a meta-analysis of studies assessing visuospatial performance under incidental and intentional acquisition conditions, comparing young and older participants [8]. Studies were conducted in which incidental and intentional visuospatial memory was evaluated in young and older adults in tests involving the recall of the spatial position of several objects, with strict control on the nature of coding [6,9,10]. The ratio of performance was computed in each study and it was observed that, in all of them, older participants performed worse than younger participants, with a more pronounced deficit observed under incidental coding conditions. In addition, the authors performed two experiments assessing spatial memory for objects in real life, contrasting incidental and intentional coding and age; the results supported the higher impairment in incidental coding with age [8].

According to the previous findings, visuospatial performance is highly vulnerable to aging, and this vulnerability is more pronounced when information is incidentally acquired. Subsequent studies have shown that the difficulty of the tasks and the modality of information coded influence the degree of decline with age, where the more difficult tasks (e.g., n-2 back vs. n-1 back) and visuospatial learning (compared with verbal) are the most sensitive [11]. In accordance, it was observed in different works that the decline in visuospatial incidental ability starts during a person’s fifties and becomes more pronounced in their sixties [8,11,12,13]. Together, these findings indicate that a test to assess the visuospatial incidental association of information could be a sensitive tool for detecting earlier age-related changes in healthy adults. To test this, Lopez-Loeza, et al. [13] evaluated incidental visuospatial memory and found a decrease in performance even in healthy adults aged 45–65 years when compared with young adults (18–40 years old), suggesting a high sensitivity of evaluations focused on incidental visuospatial coding for subclinical changes in cognitive ability.

Episodic memory depends on the temporal lobe structure and function; the successful codification of episodic memory is associated with the activation (measured with fMRI) of the hippocampus and temporal lobe cortices during the study of the information [14,15]. In addition, the activation of the hippocampus and prefrontal cortex was associated with associative memory retrieval [15].

The higher decline for incidental versus intentional coding with aging suggests that different neural substrates or circuits underlie the two forms of coding. In this sense, Rugg et al. (1997) compared the incidental and intentional coding of verbal information and observed the activation of the left hippocampus without differences in the type of coding [2]; however, intentional coding induced higher prefrontal and parietal activation compared with the incidental mode. Stark and Okado [16] observed that successful intentional and incidental coding was related to the activation of the hippocampus, entorhinal cortex, and para-hippocampal cortex. Additionally, in an incidental memory task in which the participants were attending the distinctiveness of the stimuli, Carr et al. (2013) [17] observed the higher activation, measured with fMRI, of CA1 and subiculum when the participants coded and successfully recalled stimuli. Moreover, hippocampus and para-hippocampal cortex activity is required for the association of objects and their positions [18]. These findings suggest that both types of coding are dependent on temporal lobe activity, implying subtle differences in the circuits supporting each one.

It has been reported that first-degree descendants of patients with Alzheimer’s disease (DAD) show changes in the neural substrate early in adulthood. Reduced gray matter in the right precuneus, reduced activation in the mesial temporal lobe and fusiform gyrus, and minor hippocampal activation have been reported [19,20,21]. Lampert, et al. [22] measured the integrity of the neural substrate in DAD participants through magnetic resonance imaging to search for atrophy markers and followed their progress over 4 years [22]. The results showed a significantly higher progression of atrophy in the entorhinal cortex and hippocampus in the DAD compared with NoDAD participants; however, this progression was linked to the APOe4 genotype. Additionally, the DAD participants showed a higher cognitive decline over a 4-year period. A comparison of DAD participants with early-onset and late-onset parents with control subjects in a test–retest study also revealed a decline in both DAD groups after a follow-up period of 4 years [23]. However, Donix, et al. [24] reported lower scores in processing speed, executive function, and delayed memory when comparing DAD to NoDAD participants without differences in the APOE4 genotype.

The cognitive abilities of descendants of Alzheimer’s disease patients (DAD) have also been assessed in other studies, where the main findings show that DAD participants have a general cognitive impairment, as well as specific impairments in attention, episodic memory, verbal memory, working memory, and visuospatial memory [25,26,27]. Regarding the latter, long-term visuospatial memory deficiencies were observed in tests such as the delayed recall component of the Visual Reproductions (VR-d) [28], Rey Osterrieth Com-plex Figure delayed recall [24], and the doors and people test [29]. In these studies, no dis-tinction was made regarding the severity of visuospatial memory decline with respect to different cognitive deficiencies.

In the present study, we aimed to determine whether visuospatial incidental/intentional memory shows a decline in middle-aged (35–55 years old), cognitively healthy DAD participants. We hypothesized, based on the aforementioned findings, that DAD participants would have lower accuracy in a test requiring incidentally acquired visuospatial relational information, with a minor benefit of intentional learning. Based on the results showing that young and aged participants had better performance under intentional rather than incidental coding conditions, we assessed the benefit to the performance that occurred when intentional effort was applied to learn the same information. With this objective, the incidental/intentional visuospatial memory of 55 DAD participants was characterized and compared with that of 55 paired NoDAD participants.

## 2. Materials and Methods

A cross-sectional, observational, and analytical study was carried out. Inclusion criteria: The participants were male and female (*n* = 55, group DAD) first-degree descendants of 22 men (40%) and 33 women (60%) who were patients with Alzheimer’s disease. Only first-degree relatives of one parent who was diagnosed with AD were taken into account. Male and female individuals without a familial antecedent (*n* = 55, group NoDAD), paired by age and gender with the DAD group as much as possible, were also included. The participants were men and women aged 35–55 years who were right-handed, with normal or corrected visual acuity. All the participants were invited from the Unidad de Medicina Familiar 84 (UMF 84) Tacícuaro, Michoacán, from the Instituto Mexicano del Seguro So-cial (IMSS). All procedures were performed in accordance with the Ethics and Research Committee of the Instituto Mexicano del Seguro Social and in accordance with the Decla-ration of Helsinki. This study was approved by the Ethics and Research Committee 1602 from the Hospital General de Zona # 1, IMSS, with approval number 2022-1602-054. The clinical archive was reviewed after receiving authorization from the Ethics and Research Committee and the UMF 84 Directives, and phone calls were made to first-degree relatives of Alzheimer’s disease patients diagnosed by the neurology department. The diagnoses of the parents were performed by neurologists with respect to the second level of attention, following the algorithm of the hospital. No genetic studies were performed involving the parents; the diagnosis was clinically based. Similarly, control participants were personally invited from the family medicine service when attending medical appointments or as companions. Each participant gave informed written consent.

The following demographic data were registered: sex, age, occupation, civil state, schooling years, position in the birth order, arterial pressure, body mass index, glucose levels, and comorbidity with diabetes and hypertension. Raven’s progressive matrices (RPM) test, adapted to the Spanish population [30], was applied to assess visuospatial intelligence and to obtain each participant’s IQ ranges to assess whether the IQ and visuospatial reasoning influence performance in the visuospatial incidental/intentional test. The Hamilton Anxiety Scale adapted to the Spanish population [31] was used along with the Beck Depression Inventory adapted to the Mexican population [32] to assess the motivation–emotional state of the participants. Additionally, the Folstein mini-mental state test (MMSE) adapted to the Spanish population with a rating of 35 points [33,34] was administered to the participants. Exclusion criteria: Participants with moderate to severe depression, with values under 24 in the MMSE, with systemic hypertensive disease, antecedent of cranioencephalic damage with unconsciousness, or neurological disease referred to in the interview were excluded from the study.

**Figure 1:**
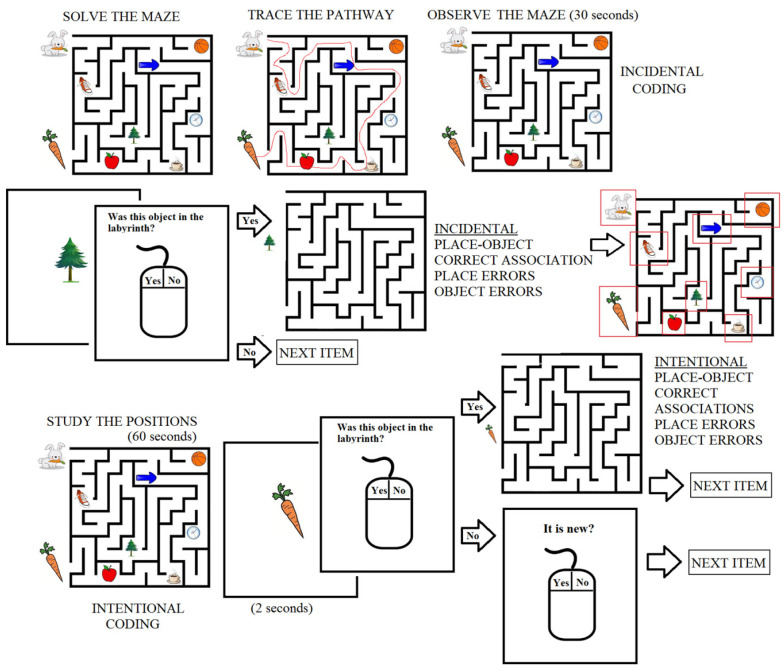
Diagrammatic representation of the incidental/intentional visuospatial test. The red square indicates the area considered as the correct position for each object. Modified from Junco-Muñoz et al. 2024 [12].

The visuospatial incidental/intentional memory test was designed at the Laboratory of Clinical and Experimental Neurophysiology, whereas the software was designed at the Instituto Tecnológico de Morelia and run on a laptop [12,13,35,36]. Briefly, a labyrinth containing 8 objects of common use was presented to the participants at the start of the test along with the instructions to mentally solve it. After that, the participants traced the route, and were given 30 s to observe the labyrinth without receiving any additional instructions (Figure 1).

Then, an object was presented at the center of the screen for 2 s, followed by the question “Was this object in the labyrinth?” If the participant answered “YES”, the labyrinth, now devoid of objects, was presented, and the participants had to drag the object to the respective position from memory. After that, the next item was presented. If the participant answered “NO”, the next object was presented. Following this, 7 new objects were interspersed with the eight objects presented in the labyrinth, and the participants had to respond based on their incidentally acquired memory. Once the participant had responded to the questions for all 15 objects, the labyrinth with the 8 objects was presented to the participant once again with the instruction to memorize the positions of the objects. After 60 s, an object was presented on the center of screen for 2 s, followed by the question “Was this object in the labyrinth?” If the participant answered “YES”, then an empty labyrinth was presented, and the object would need to be placed in the respective position from memory. If the participant answered “NO”, they were asked the following question: “Is the object new?” The participant was required to remember whether the object was new or part of the set of 7 objects previously used during the first incidental test as a contrast object to the 8 objects in the labyrinth.

The number of correct object–position associations (an object placed in its correct position in the labyrinth), object errors (when a new object was placed into the labyrinth), and place errors (when an object presented in the labyrinth was placed in an erroneous position) for each condition (incidental and intentional) were obtained and compared. As these scores did not present a normal distribution, a between-group comparison was per-formed using the Mann–Whitney U test. Comparisons between conditions (incidental and intentional) in each group were also performed using Wilcoxon’s test, in order to assess whether the participants’ performance under incidental coding conditions was similar to their intentional coding accuracy. Finally, a multiple correlation analysis, which considered sex as a partial independent factor and schooling years as a partial dependent factor, was conducted between memory scores and age for each group of participants in order to determine any potential decline related to age.

As mentioned in the Introduction, associated information recall declines with age more than individual item recall, and the deficit is higher under incidental compared to intentional coding conditions. The participants were thus classified as high- or low-accuracy performers for each coding condition based on the number of correct object–position associations. Under incidental conditions, participants were considered highly accurate if they were able to identify 4–8 correct object–position associations; in the intentional codification, participants who determined 6–8 correct object–position associations were classified as highly accurate. The number of total place–object associations included in the test (8) was based on the mean number of items in the short-term memory span (7 to numbers or words) [36,37], whereas the ranges for accuracy were set based on the memory span reported for geometric figures and the visuospatial span reported in the Corsi block-tapping task [38,39], with a mean of 5 items maintained in memory under normal conditions. We also based the scores on the performance of the participants assessed by our group in previous studies, in which young adults had no problem recalling 6 to 8 items, and on the distribution of the scores for place–object associations in previous works [12,13,40]. Once classified, the frequency distributions for the participants’ accuracy in both coding conditions, accuracy in incidental but not in intentional coding, accuracy in intentional but not in incidental coding, and accuracy in both coding conditions were compared between groups with the chi^2^ test. After the accuracy classification, a mixed-model design analysis was performed to explore the factors significantly affecting the accuracy, in which the familial antecedent, IQ (based on Raven’s test performance to assess whether visuospatial ability influences the accuracy), sex (in order to evaluate whether sexual differences exists in the performance of the test), anxiety levels, and comorbidity (because metabolic disease is related to reduced accuracy in the present test [35]) were considered as fixed effects (i.e., variables possibly explaining the accuracy level). The individual influences of these fixed effects on accuracy and the influences of interactions between the familial antecedent and the other fixed factors were included in the model. Age, years of schooling, glucose levels, and arterial pressure were considered aleatory effects (effects that influence but not determine the performance). Statistical analysis was performed using Systat 12 and GraphPad 5.03; a statistical value of *p* < 0.05 was considered significant.

## 3. Results

### 3.1. Clinical and Demographic Data

A description of the characteristics of the participants of each group is shown in Table 1. No differences were observed between groups concerning age, years of schooling, MMSE and RPM scores, the frequency of obesity and overweight, sex distribution, and comorbidity with diabetes. Participants with controlled diabetes were included in the study based on previous works where no deficiency in incidental memory was observed in controlled diabetic patients [35,40], whereas hypertensive patients showed deficiencies in this test in relation to the control of pressure and medication (controlled patients receiving angiotensin II receptor blockers were better than patients receiving angiotensin-converting enzyme inhibitors) [36]. There were no significant differences in the frequency distribution of anxiety levels between groups (chi-squared = 0.077, df = 1.000, *p* = 0.781). There were 47 participants in the NoDAD and 48 in the DAD groups with absent anxiety, while 7 in the NoDAD and 8 in the DAD groups presented a low anxiety level. Depression levels were similar among the groups, with chi-squared = 1.009, df = 1.000, and *p* = 0.315. The participants in the NoDAD group showed minimal signs of depression, whereas, in the DAD group, one participant showed moderate signs of depression and all other participants showed minimal signs of depression.

### 3.2. Incidental/Intentional Visuospatial Test

The test was applied to a different sample consisting of 219 18–85-year-old (58.97 ± 13.88) adults (187 female; educational level: 5.3% no formal, 44.0% basic, 19.6% secondary, 20.4% high school, 10.7% graduate). Cronbach’s test was performed for all components of the test, namely, recognition questions related to the object in the labyrinth and recall questions related to place–object associations, both incidental and intentional. The Cronbach’s α value obtained was 0.798, ω = 0.772. Cronbach’s alpha was additionally computed only for place–object association questions due to the higher sensitivity of this information, which was used to classify the accuracy of the participants. The inclusion of both incidental and intentional responses resulted in a value of 0.812, with a ω = 0.815. An exploratory factor analysis (EFA) with promax rotation revealed a latent structure consistent with the underlying cognitive processes assessed. In the recognition phase, a four-factor solution emerged, accounting for 38.7% of the total variance, with factors organized according to the encoding conditions (incidental vs. intentional) and semantic characteristics of the stimuli. In the recall phase, a more parsimonious two-factor structure was identified, explaining 38.4% of the variance, clearly differentiating between intentional encoding (associated with strategic cognitive processing) and incidental encoding (related to spontaneous retrieval mechanisms). These findings support the structural validity of the task and its sensitivity to distinguish distinct visuospatial memory processes.

**Figure 2:**
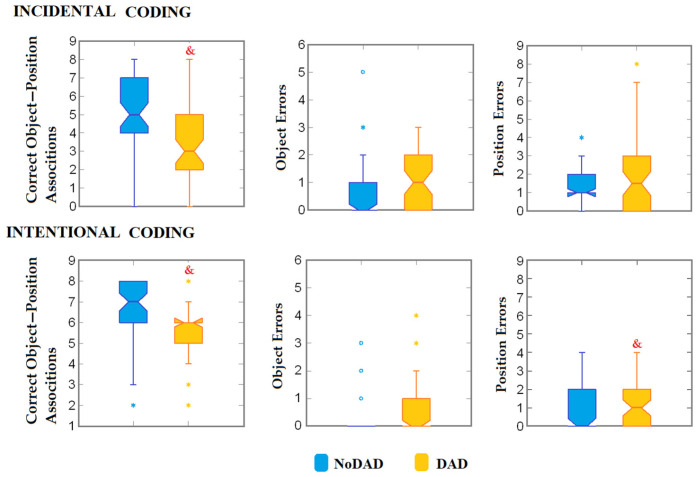
Intergroup comparison of memory variables under incidental and intentional coding. Box plots represent median ± interquartile ranges, and notch constriction represents the approximate 95% confidence interval around the median. *°, Out of range values. &, NoDAD vs. DAD; *p* < 0.05. DAD participants scored significantly lower number of correct object–position associations under both incidental and intentional coding conditions. In addition, DAD participants showed higher number of position errors.

The comparisons of memory performance showed significant differences between groups. The DAD group had a significantly lower number of correct place–object associations under incidental coding (U = 2183.00, *p* < 0.001, η2 = 0.146, d_Cohen_ = 0.827) and intentional coding (U = 2062.00, *p* = 0.001, η2 = 0.098, d_Cohen_ = 0.66) conditions. No differences in object errors (U = 1291.00, *p* = 0.147, η2 = 0.016, d_Cohen_ = 0.255) or position errors (U = 1363.50, *p* = 0.359) were observed under incidental coding conditions. Under intentional coding conditions, the DAD group showed a higher number of position errors (U = 1125.50, *p* = 0.015, η2 = 0.049, d_Cohen_ = 0.452) than the NoDAD group, whereas no differences were observed in object errors (U = 1310.50, *p* = 0.133, η2 = 0.007, d_Cohen_ = 0.232) (Figure 2).

**Figure 3:**
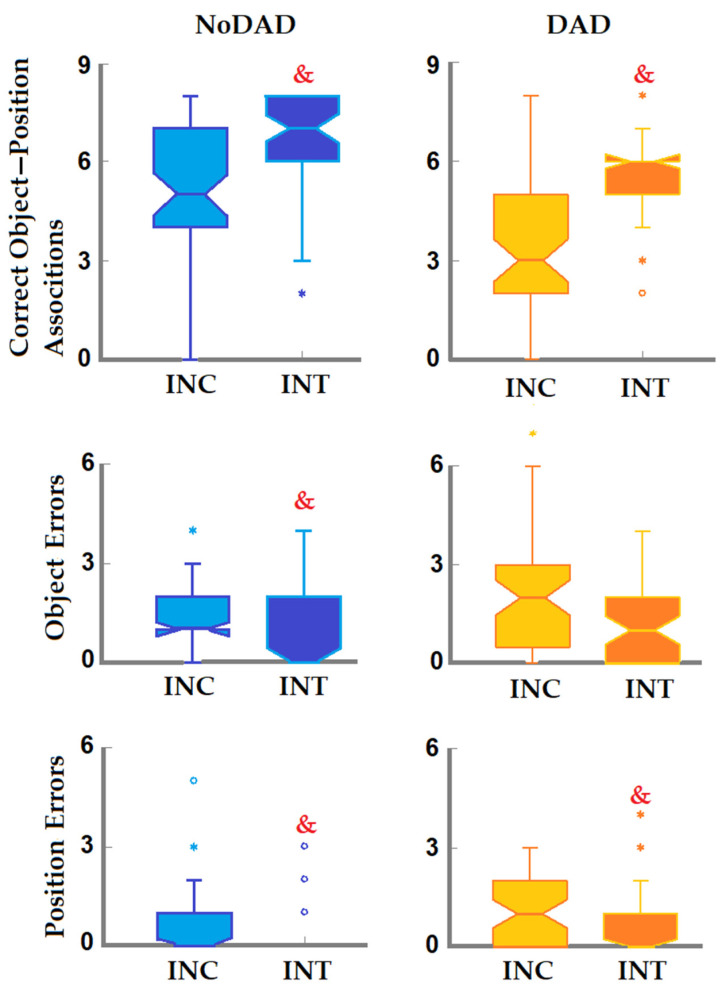
Intragroup comparison of memory scores. Incidental versus intentional performance was compared for each group. Box plots represent median ± interquartile ranges, and notch constriction represents the approximate 95% confidence interval around the median. *°, out of range values; &, incidental vs. intentional performance; *p* < 0.05. NoDAD participants demonstrated a higher number of correct object–position associations and a reduced number of object and position errors after the intentional study, whereas the DAD group participants also increased the correct object–position association and reduced position errors.

The intragroup comparisons showed significant differences between the incidental and intentional performance of the NoDAD participants (Friedman = 197.142, *p* < 0.001). Paired comparisons revealed a higher number of correct object–position associations under intentional coding conditions (z = 5.151, *p* < 0.001, d_Cohen_ = 1.931) and a lower number of object (z = −2.087, *p* = 0.037, d_Cohen_ = 0.587) and position errors (z = −2.498, *p* = 0.013, d_Cohen_ = 0.715) under intentional conditions compared with incidental coding (Figure 3).

The DAD group also demonstrated a significant difference between incidental and intentional performance (Friedman = 125.003, *p* < 0.001). Although the DAD group exhibited a lower number of correct object–position associations in the intergroup comparison, the participants showed an increase in correct responses under intentional conditions compared with incidental coding (z = 5.84, *p* < 0.001, d_Cohen_ = 2.55). In addition, the DAD group exhibited a significant reduction in object errors (z = −1.989, *p* = 0.047, d_Cohen_ = 0.557) but not in place errors under intentional conditions (z = −1.516, *p* = 0.130, d_Cohen_ = 0.418) (Figure 3).

**Figure 4:**
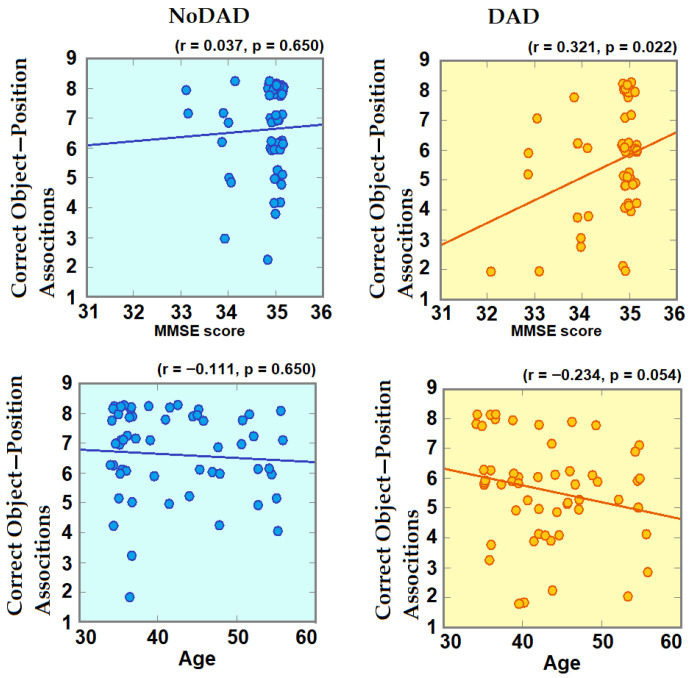
Pearson correlation of correct object–position responses under intentional coding conditions with MMSE scores and age. Multiple correlation test with school years and anxiety scores as partial dependent variables and sex as a partial independent variable was performed. *p* values were obtained with Bonferroni adjustment, the line represents linear smoothing. DAD participants showed a significant positive correlation between correct object–position responses and MMSE scores and a bias to a minor number of correct object–position associations with age.

Multiple correlation tests of the behavioral variable with age and MMSE scores were carried out for each group, considering sex, obesity, and anxiety as partial independent variables and schooling years as a partial dependent variable. A significant positive correlation was observed between the number of correct object–position associations under intentional conditions and the MMSE scores, but only within the DAD group (NoDAD: r = 0.032, *p* = 0.659; DAD: r = 0.321, *p* = 0.022; q_Cohen_ = 0.296); higher MMSE scores were as-sociated with a higher number of correct responses only in DAD participants. In addition, a negative correlation between the number of correct object–position associations under intentional conditions and age was marginally significant for the DAD group but not for the NoDAD group (DAD: r = −0.234, *p* = 0.054; NoDAD: r = −0.111, *p* = 0.650, q_Cohen_ = 0.127) (Figure 4). No significant correlations were observed for incidental behavioral variables and age or MMSE scores.

### 3.3. Classification of Participants by Performance

Once the participants were classified according to the number of correct object–position associations, a significant difference in efficiency was observed. The DAD group had significantly higher percentages of low-accuracy members for incidental conditions (72.72% vs. 40% of the NoDAD group) and intentional conditions (41.81% vs. 20% of the NoDAD group). In addition, the number of participants with low-accuracy performance in both incidental and intentional conditions was higher for the DAD group (36.364%) than for the NoDAD group (16.364%, Mantel–Haenzel X^2^ = 11.197; *p* = 0.001; d_Cohen_ = 0.673) (Table 2).

Finally, a mixed-model analysis was performed in order to evaluate the factors related to the accuracy performance of the participants. The fixed factors were group, sex, anxiety, Raven IQ category, and the interaction of the familial antecedent with other fixed factors. The aleatory factors were age and years of schooling, glucose levels, and arterial pressure. Under incidental codification conditions, the results showed a significant effect for the interaction of anxiety and familial group (F = 3.928, *p* = 0.05). The comparison of frequency distribution was significant (Mantel–Haenszel statistic = 3.981; Mantel–Haenszel chi-square = 10.415, *p* = 0.001; d_Cohen_ = 0.646), and the 2×2 chi-squared tables showed a significant difference in the number of high-accuracy participants depending on anxiety levels. Ninety-five participants exhibited absent anxiety levels, of which the NoDAD participants scored 29.47% in high-accuracy and 20% in low-accuracy performance. In contrast, only 12.63% of DAD participants had high-accuracy performance, while 37.89% had low-accuracy performance. No differences were observed in the participants with high levels of anxiety. No specific factors were related to efficiency under intentional coding conditions.

## 4. Discussion

The results show a low accuracy in coding visuospatial relational information under both incidental and intentional conditions among first-degree descendants of AD patients. This deficient performance was evident in the low number of correct responses achieved by the DAD group under the two coding conditions, in the high number of low-accuracy participants under incidental coding conditions, and in the high number of participants with low accuracy in both coding conditions. Together, these results indicate an impairment in visuospatial performance related to the familial history of AD, seen earlier (33–55 years old, mean age in the 40s) than the age-related decline in NoDAD, which has been reported to begin in the age range of 50–60s [8,11]. Compared to the DAD group, NoDAD participants had a higher proportion of high-accuracy performers under both incidental and intentional conditions. The proportion of participants whose performance was accurate under intentional conditions amounted to 80% for the NoDAD group in contrast to 58% for the DAD group, whereas the percentage of participants whose performance was accurate under incidental coding conditions was 60% for NoDAD and 27.27% for DAD participants. Furthermore, the proportion of participants who showed low-accuracy performance under incidental conditions and were unable to improve under intentional effort conditions was only 16% for the NoDAD group and 36% for the DAD group. In accordance with the present results, one study previously reported reduced spatial memory abilities in first-degree descendants of AD patients; this effect was observed after evaluating a large sample of participants (4086) in a meta-analysis [26]. In contrast, other studies examining small samples of DAD reported subtle changes in cognitive performance. La Rue, et al. [23] reported subclinical changes in a series of tests conducted with intervals of 4 years among relatives of AD patients, including those with early- and late-onset AD parents. In addition, Hom, et al. [27] evaluated 20 DAD and 20 non-DAD participants in terms of intelligence, memory, and verbal abilities, and found a lower performance in descendants together with a higher percentage of deficient participants (50% compared to 20% in non-descendants). The present results show a marked decline in a relatively small sample, allowing for the characterization of the participants as either low-accuracy or high-accuracy performers, and enabling us to pay special attention to those participants who showed a low accuracy under both incidental and intentional coding conditions. The potential link between the increased frequency of low-accuracy performers and the risk of developing AD cannot be addressed with the present results; longitudinal studies of DAD participants are needed to follow the changes in performance in tests evaluating similar attributes (i.e., incidental/intentional visuospatial information) with aging. An additional group of interest in the study comprises participants who showed high accuracy under incidental coding conditions but low accuracy under intentional coding conditions. They appear to have had difficulties when the cognitive sources were directed toward learning, and their performance was impaired only when attention was given to the task. Additionally, the proportion of these participants was similar in both NoDAD and DAD participants (reaching 10% of the total), indicating no relation to the family history of AD. However, sustained attention was assessed in the early stages of AD, where a deficit in attention was reported when it was evaluated using the sustained attention to response task, which correlated to the MMSE score [41]. Early changes in cognitive processes requiring sustained attention could thus be related to the early stages of AD. In the present work, these participants who showed a poor performance under intentional effort conditions of coding after responding with a high accuracy under incidental coding conditions were affected in terms of coding ability when sustained attention was required; however, this needs to be evaluated in future work along with the possible relation between different levels of performance and the APOE4 genotype.

We determined from the present results that both incidental and intentional visuospatial coding are reduced in first-degree descendants of patients with AD. This decline was detected before the emergence of significant changes in tests such as the MMSE and RPM, suggesting the higher sensitivity of visuospatial relational tests in detecting subtle cognitive decline. The MMSE and MoCA are the principal tests used to evaluate the stage of deterioration in AD, although a higher sensitivity for the MoCA has been reported at earlier stages. The combination of the two tests was found to be a highly accurate indicator of posterior AD progression. The CANTAB test pairs associate learning (a test of visuospatial associative learning) and a graded naming test (a test of semantic memory), showing a very high sensitivity for the detection of AD progression [42,43]. In this regard, adult participants in previous works were evaluated using the present visuospatial test with normal MMSE and MoCA values as the inclusion criteria, and, despite these normal values, they were found to have low accuracy in the test [12]. All the participants in the present work had MMSE values above the limit for cognitive deterioration, and a proportion of the participants showed low accuracy in one or two coding conditions. According to the psychobiological phenomenon, a moderate reduction in memory buffer capacity, which can be normal in older people, is unusual in young people. The presence of only 0–2 associations in an elderly individual indicates a significant decline in memory; however, in a young person, this performance is a sign of alarm. The method used in this work is an initial neuropsychological approach, created with the aim of detecting early changes in cognition in the adult population that are greater than expected due to aging and possibly related to the neurodegenerative process. More specifically, neurodegenerative disorder detection tests such as the presented approach could be employed as screening tools which are capable of revealing early changes in cognition, enabling the development of strategies focused on modifiable risk factors.

Incidental and intentional coding are related to the activity of temporal lobe structures [2,15], which could be compromised earlier in DAD participants, as well as in NoDAD participants at risk of developing AD. Several early changes occur in the cerebral physiology associated with AD, and different changes in the neural tissues and function have been reported in cognitively unimpaired first-degree descendants of individuals with AD. In this regard, a reduction in the thickness of gray matter in the entorhinal cortex, subiculum, and temporal lobe-associated cortex has been reported in cognitively healthy family members of patients with AD. This reduction was independently related to a family history and the presence of APOE4, but was additive in participants carrying both characteristics [19,24]. Similarly, a reduced thickness of gray matter was reported in the right precuneus of participants with a family history of AD. In contrast, when APOE4 was also present, the reduction was observed bilaterally at the insula [21]. Ramirez-Torano, et al. [44] assessed non-cognitively impaired descendants of AD patients 10 years before the typical onset age of sporadic AD. The authors observed a reduced connection between the posterior and temporal superior areas and between the posterior areas and the amygdala in descendants, which was not observed when considering the presence of APOE4, cognitive punctuation in neuropsychological scales, sex, age, or education level. Thus, Johnson, et al. [20] compared the activation of the temporal lobe structures of DAD participants and unrelated participants, who were carriers and non-carriers of APOE4, through a recognition memory task. Greater activation in response to novel items was observed in NoDAD participants in the fusiform gyrus, hippocampus, and amygdala, whereas no differences were observed among carriers and non-carriers of APOE4. When the interaction between family history and APOE4 was compared, a higher activation of the hippocampus was observed in NoDAD–APOE4-positive participants compared with DAD–APOE4-positive carriers. The participants in the study were cognitively healthy, and the mean age of the participants was in the fifties. In the present work, the participants ranged between 35 and 55 years old and showed deficient visuospatial learning, in agreement with the results reported by Ramos, et al. [26].

Changes in the neural substrate of the participants could thus have already occurred in their early years but remained undetectable through clinical neuropsychological tests. According to this view, the negative correlation between age and the number of correct associations reached under intentional coding conditions only observed in DAD participants—though marginal in significance—is an interesting result which needs to be validated in future research in order to determine whether this is indicative of an earlier age-related detriment. Longitudinal studies evaluating visuospatial incidental/intentional memory in DAD must be performed to follow the age-associated decline in this population and assess the possibility of an accelerated decline with age.

Neuropsychological tests are cheap and easy to apply and, given the impossibility of performing clinical reviews with imaging diagnoses or genetic characterization for the entire population, these tools can serve as a filter to redirect clinical resources to vulnerable populations after a preliminary assessment. In addition, early detection allows the therapeutic window to be broadened to assess both pharmacological and non-pharmacological strategies in order to preserve the cognitive status of the population for the longest possible period. The 2024 report by the Lancet Commission identified hypertension, obesity, alcohol consumption, diabetes, smoking, depression, social isolation, and a low level of physical activity as modifiable risk factors accounting for the prevention of 40% of dementia cases [45]. All these factors can be addressed through affordable interventions directed toward vulnerable populations, such as older adults and DAD, and their impact on the progression of cognitive decline would be higher when initiated earlier. The early detection of deficiencies, as reported here, would contribute as an initial tool to be applied in special programs for participants with very low scores in the visuospatial incidental/intentional test, which can be considered as a starting point toward the optimal use of limited public health resources.

Although the use of the same information under incidental and intentional coding conditions in the present test may raise doubts about the specificity of the evaluated encoding process, the instructions and the interference tests (to solve the labyrinth) reduce the need for conscious efforts to learn the objects’ positions under incidental coding conditions. The participants also expressed surprise when the question about the objects appeared in the test. As a general practice, observing the reaction of the participants when the incidental questions are presented gives the researcher an idea of the use of intentional coding by the participant. The specific question “Did you try to memorize the objects–positions before the instruction to do it?” can also be presented to the participants once the test is finalized. With regard to the possible contribution of incidental coding to the intentional performance, we consider that using the same information under incidental and intentional coding conditions provides valuable information about the relationship between incidental and intentional performance. Although the use of the same information leads to the possible influence of incidental coding, re-exposure, and practice effects on intentional performance, this would lead to participants with a good performance in incidental coding always performing well in intentional coding. The present results indicate that successful incidental coding is not always associated with successful intentional coding and that the two coding conditions may present different levels of performance, supporting differential processing in the temporal lobe circuits. Thus, this design offers the possibility to analyze the relationship between incidental and intentional coding, taking into account the limitations and advantages of using the same information.

The absence of biological markers to identify AD risk according to the APOE genotype in the participants and the parents of the participants is a limitation of the present study; however, the diagnoses were performed by neurologists with experience based on clinical criteria and following the algorithm and procedures of the institute. Future work including APOE characterization and its impact on memory assessed through the visuospatial incidental/intentional test employed in this study must be performed, as well as research focused on the interaction between a family history of AD and APOE characterization. Additionally, our sample was geographically restricted as the participants were all invited from UMF 83 and limited to one community (Tacícuaro, Michoacán). Although both the DAD and NoDAD participants belonged to the same community, their characteristics may differ from the other populations in cities with different socio-economic conditions. Finally, the current test requires standardization in several centers and different geographical regions, along with more comprehensive neuropsychological assessments, to support its sensitivity in pre-clinical stages.

## 5. Conclusions

The presented results indicate an impairment in visuospatial incidental/intentional performance related to the familial history of AD, which was seen earlier (35–55 years old, mean age in the 40s) in adulthood.

## Figures and Tables

**Table 1:** Characteristics of the samples.

	NoDAD			DAD			
Number of cases	55 (38 women)			55 (36 women)			t, *p*, d_Cohen_
Age	41.964 (7.288)			43.127 (6.594)			−0.878, 0.764, −0.167
Years of Schooling	15.600 (4.349)			15.873 (4.308)			−0.330, 0.742, −0.063
MMSE	34.800 (0.487)			34.655 (0.700)			1.265, 0.208, 0.241
RPM	Low–middle–high 7–35–13			Low–middle–high 4–36–15			X^2^, *p*, d_Cohen_ 0.975, 0.614, 0.189
Obesity	1	2	3	1	2	3	4.681, 0.096, 0.421
	38	13	4	32	11	12	
Diabetes	6			1			3.814, 0.051, 0.379

NoDAD, group of non-descendants of AD patients; DAD, group of descendants of AD patients. Values of age, years of schooling, and Folstein’s mini-mental examination (Spanish version) (MMSE) scores are expressed as mean ± standard deviation. t, *p*, and d_Cohen_ represent values for these variables. Raven’s progressive matrices (RPM) test values: low, middle, and high ranges of IQ after conversion from ranges of RPM test. Obesity: 1, normal weight; 2, overweight; 3, obesity. Diabetes: number of participants diagnosed with diabetes. chi^2^, *p*, and d_Cohen_ values are presented for these categorical variables.

**Table 2:** Frequency distribution of high- and low-accuracy participants under incidental and intentional coding conditions per group.

**NoDAD**		**Intentional Coding**		
		High accuracy	Low accuracy	Total
Incidental coding	High accuracy	31 (56.364%)	2 (3.636%)	33 (60.000%)
	Low accuracy	13 (23.636%)	9 (16.364%)	22 (40.000%**)**
	Total	44 (80.000%)	11 (20.000%)	55 (100.0%)
**DAD**		**Intentional Coding**		
		High accuracy	Low accuracy	Total
Incidental coding	High accuracy	12 (21.818%)	3 (5.455%)	15 (27.273%)
	Low accuracy	20 (36.364%)	20 (36.364%)	40 (72.727%)
	Total	32 (58.182%)	23 (41.818%**)**	55 (100.0%)

NoDAD, group of non-descendants of AD patients; DAD, group of descendants of AD patients. Values are frequencies and percentages. Mantel–Haenzel X^2^ = 11.197; *p* = 0.001; d_Cohen_ = 0.673. The NoDAD group shows a higher percentage of participants with high accuracy in both coding conditions, whereas the DAD group had higher percentage of participants with low accuracy in both coding conditions.

## Data Availability

The data presented in this study are available on request from the corresponding author due to restrictions (permissions must be authorized by the Mexican Institute of Social Security).

## Abbreviations

The following abbreviations are used in this manuscript:

DAD	Descendants of Alzheimer’s disease patients
NoDAD	No descendants of Alzheimer’s disease patients
RPM	The Raven’s Progressive Matrices Test—three-letter acronym
VR-d	Delayed recall component of the Visual Reproductions
UMF	Family medicine unit
MMSE	Folstein’s mini-mental state examination
